# Fostering engagement in virtual anatomy learning for healthcare students

**DOI:** 10.1186/s12909-024-05278-5

**Published:** 2024-04-16

**Authors:** Lauren Singer, Lily Evans, Daniel Zahra, Ifeoluwa Agbeja, Siobhan Moyes

**Affiliations:** Peninsula Schools of Medicine and Dentistry, Faculty of Health, University of Plymouth, Plymouth, UK

**Keywords:** Anatomy, Virtual learning, Virtual anatomy, Technology acceptance model, Integrated curriculum, Technology enhanced learning

## Abstract

**Background:**

The use of virtual learning platforms is on the rise internationally, however, successful integration into existing curricula is a complex undertaking fraught with unintended consequences. Looking beyond medical and pedagogic literature can provide insight into factors affecting the user experience. The technology acceptance model, widely used in software evaluation, can be used to identify barriers and enablers of engagement with virtual learning platforms. Here, the technology acceptance model was used to scaffold the exploration of the factors that influenced students' perceptions of the virtual anatomy platform, Anatomage and how these shaped their intention to use it.

**Methods:**

Focus groups identified factors influencing students use of the Anatomage tables. Interventions were rolled out to address these findings, then further focus groups and the technology acceptance model identified how factors including self-efficacy, enjoyment, and social norms influenced students’ intention to use the Anatomage table in the future.

**Results:**

Students raised significant concerns about understanding how to use the Anatomage table. Moreover, students who considered themselves to be poor at using technology perceived the Anatomage table as more complicated to use. The subjective norm of the group significantly altered the perceived ease of use and usefulness of the Anatomage. However, enjoyment had the greatest impact in influencing both perceived usefulness and perceived ease of use. Indicating that enjoyment is the largest contributing factor in altering technology engagement in healthcare cohorts and has the biggest potential to be manipulated to promote engagement.

**Conclusions:**

Focus groups used in tandem with the technology acceptance model provide an effective way to understand student perceptions around technology used in the healthcare curricula. This research determined interventions that promote student engagement with virtual learning platforms, which are important in supporting all healthcare programmes that incorporate technology enhanced learning.

**Supplementary Information:**

The online version contains supplementary material available at 10.1186/s12909-024-05278-5.

## Introduction

In the rapidly evolving landscape of education, the integration of virtual learning platforms has become instrumental in shaping the academic experience for students. As educational institutions strive to harness the potential of technology to enhance learning outcomes, a critical challenge emerges: how to effectively engage students in the utilization of virtual learning platforms. Regarding medical education, virtual anatomy platforms have become a learning modality that provides an array of accessible new learning resources [1–4]. As such, there is an expectation to integrate them into existing curricula. As with any curricular intervention, it is important to understand the barriers and enablers of using these technologies to optimise student learning.

Studies have identified that students have a strong preference for learning with digital technology, so facilitating and enabling its productive use is important [5–7]. While many students could be considered digital natives, this is not universal for all cohorts, or for the staff leading these programmes. As such, a structured approach to integration can support effective use and minimise the barriers that can limit engagement with virtual anatomy.

Peninsula Medical School runs an integrated, spiralled, enquiry-based learning (EBL) curriculum, with clinical placements throughout the 5-year programme. Due to the emphasis on self-directed study within the programme, anatomy teaching is delivered via a flipped classroom approach, using different teaching modalities including virtual anatomy, surface anatomy, medical imaging including live ultrasound, modelling and anatomical models [8–12].

An Anatomage table was purchased in 2015 to increase the range of teaching approaches and allow students access to this resource for their self-directed study. The Anatomage Table is a 3D anatomy platform for visualisation and virtual dissection. California-based company Anatomage Inc. in partnership with the Stanford Clinical Anatomy Department developed the Anatomage table to be used in healthcare education. It uses 3D digital representations of a range of human bodies, donated to The Visible Human Project® (NLM., Bethesda, USA), allowing users to dissect or build the highly detailed digital cadavers with precision. They also offer the ability to orientate cross-sectional images in different planes, which develops better spatial understanding of body structures [13].

A recent review determined that Anatomage is viewed as a useful adjunct for learning anatomy and increases student scores in anatomy assessments, with multiple papers reporting similar findings [14]. While current research demonstrates that the Anatomage is a useful tool for learning anatomy there is currently no research giving practical advice on how to effectively embed the Anatomage and similar technologies into the curriculum.

Given the evidence that our cohorts could be considered as digital natives, a light-touch training approach was taken [15]. An induction was provided to students in induction week of year 1. Students were allocated a time and group and provided with written instructions outlining the key functionality that would be used in the first sessions. A booking form was created to allow students to reserve the table for self-directed study and additional resources were provided to structure their learning. In class, students were encouraged to participate in group dissections, or virtual construction. However, the students did not engage as enthusiastically with the Anatomage table as was hoped, particularly for their self-directed learning (SDL). In response, research was undertaken to understand the barriers to using virtual anatomy platforms in the student cohorts. This led to the utilisation of the technology acceptance model to develop and implement improvements to integration, delivery, and expansion of virtual anatomy provision to medical, dental, diagnostic radiography and physician associate programmes.

**Fig. 1:**
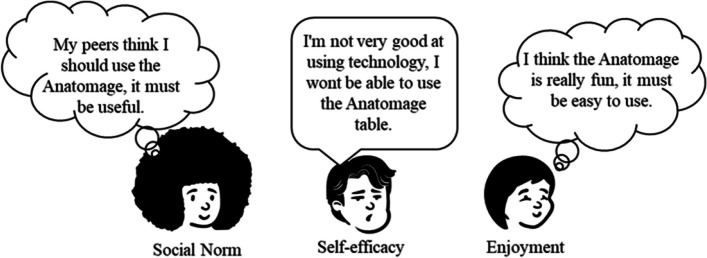
External Factors in TAM. Social norm is a user’s perception that other people think they should use a piece of equipment, and directly influences their perception of how useful the technology is. Self-efficacy is the user’s perception of how good they are at using technology and directly impacts how easy they find the technology to use. Enjoyment can influence the perception around how easy a piece of equipment is to use and how useful it is

The technology acceptance model (TAM) provides a scaffold to explain user engagement with technology (Fig. 1). While it is widely used in software evaluation, it is not a mainstay in virtual anatomy education. TAM states that there are two major factors on whether computer software will be accepted and therefore adopted by user groups. These two factors are the perceived ease of use, and the perceived usefulness in relation to their career progression [16]. Whether a piece of software is perceived as easy to use or useful is influenced by external factors. These external factors vary widely in the literature; therefore, this paper will concentrate on three commonly used external factors: self-efficacy, subjective norm, and enjoyment [17–19].

**Fig. 2:**
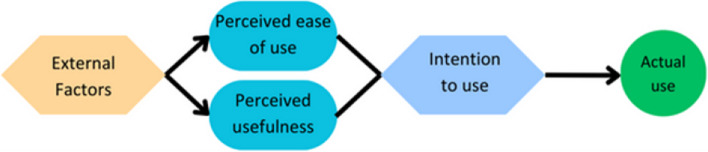
Adapted representation of TAM from Davies (1992). Multiple external variables affect a person’s perceived usefulness of a piece of technology, as well as how easy they perceive that piece of technology is to use. Perceived ease of use and perceived usefulness can then predict intention to use the piece of technology and then whether a person will actually use it

Self-efficacy is an individual’s opinion as to whether they can complete a task, or in this case, use a piece of equipment (Figs. 1 and 2). It is not an external measure of their ability to use that piece of equipment. Perceived ease of use has been shown to directly correlate with self-efficacy, meaning that people who perceive themselves as being good at using a piece of equipment will perceive the piece of technology as easier to use [17, 20, 21]. As ease of use predicts an individual’s intention to use and actual use, those with low self-efficacy are less likely to use the piece of equipment in the future.

The subjective or social norm is an individual’s perception of the extent to which they think other students think they should use a piece of equipment [18]. Social norms have been shown to directly influence perceived usefulness in the technology acceptance model [22, 23]. Students who think that other students think they should use the Anatomage table perceive the Anatomage table as more useful, meaning social norms can have a large impact on intention to use and therefore actual use of technology.

Enjoyment is an intrinsic motivator that can influence whether a person finds interacting with the technology an enjoyable experience in its own right [24]. While originally proposed as directly influencing ease of use, more recent studies have also suggested its role in altering perceived usefulness [18, 19, 25]. Furthermore, enjoyment during students’ medical degrees has been shown to have a positive impact on outcomes, suggesting an important role in student engagement and knowledge acquisition [26].

TAM has been shown to be an important model in determining and manipulating technology acceptance to encourage use in many areas, however, there is little data available exploring its use in anatomy education. Whilst a small number of studies have explored student and educator acceptance of the Anatomage table, and shown it is considered a useful tool in education [27, 28], these do not provide a theoretical basis such as the technology acceptance model as a scaffold for creating actionable ways to promote further use of the technology. Moreover, students at Peninsula did not engage with the Anatomage despite what might have been expected given this prior work. The current study was therefore designed to fully explore the barriers that students were facing at our institution through focus groups and then through quantitative data collection, based on the technology acceptance model, determine the dissonance between the previous research and our students’ perceptions. The ultimate goal of this research was to determine practical interventions to promote the use of the Anatomage to strategically embed it into the curriculum and to inform other medical schools how to effectively embed technology into their curricula in the future.

## Methods

**Fig. 3:**
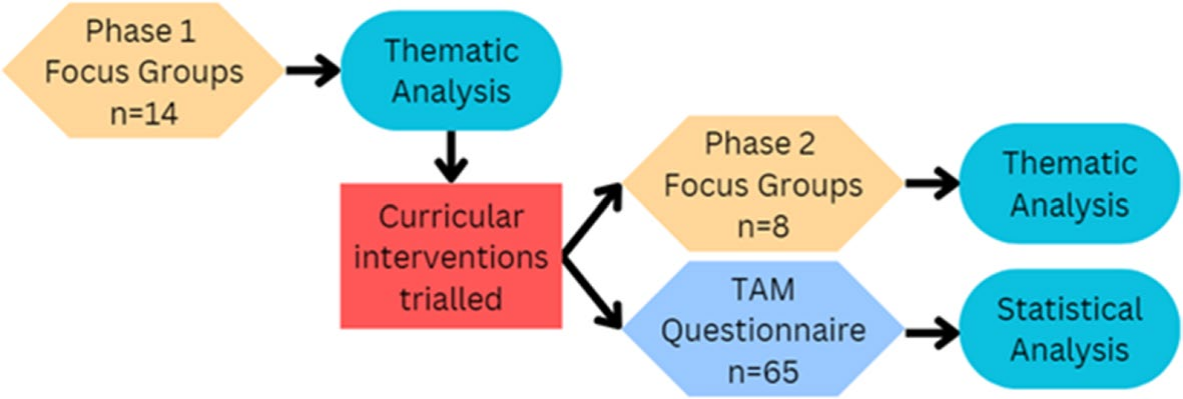
Study design and n values. This figure shows the order in which the focus groups were run and when the questionnaire was distributed

Figure 3 illustrates the study design for this research. Phase one focus groups and thematic analysis took place to understand students’ perceptions of the Anatomage system. Once themes were established, interventions were created to address these points. Phase two focus groups and the TAM questionnaire were then carried out to evaluate the impact of the interventions on students’ perceptions and explore student perceptions in more depth.

### Qualitative research

#### Participants

**Fig. 4:**
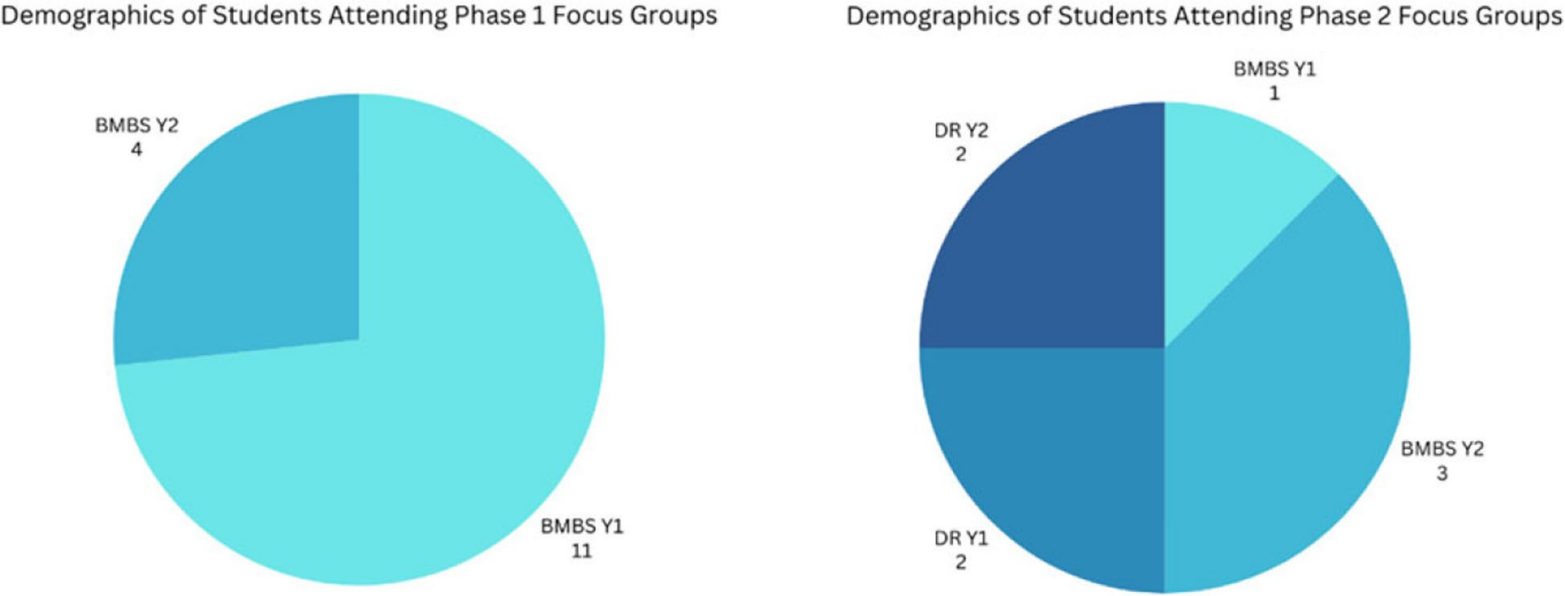
Pie charts depicting student number and programme that took part in the focus groups. In phase 1 focus groups 11 Bachelor of Medicine Bachelor of Surgery first year students took part and four second year students, 8.38% of all students who were invited to take part in the study. In comparison, in phase 2 from Bachelor of Medicine Bachelor of Surgery one year 1 student and three year 2 students took part. From the Diagnostic Radiography programme two year 1 and two year 2 students took part. This represented 1.6% of all students who were invited to take part in the study

Student volunteers were recruited from programmes within The University of Plymouth Peninsula Schools of Medicine and Dentistry that use the Anatomage tables in their anatomy curriculum. These are the years one and two students of the Bachelor of Medicine, Bachelor of Science (BMBS) programme, years one and two students of the Diagnostic Radiography (DR) programme and year one students of the Physician Associates (PA) programme. Phase 1 were recruited initially from the BMBS programme. However, covid interrupted the normal running of the curricula, so Phase 2 recruitment was delayed until face-to-face teaching resumed. Phase 2 was then recruited from all programmes that use the Anatomage. For Phase 1 the study was advertised using posters around the department, however, for phase two focus groups were advertised to students in class and via email and an online signup sheet was provided to register interest. The number of students that took part in each focus group is detailed in Fig. 4.

#### Focus groups

Data were collected through focus group discussions. These methods allowed for in-depth exploration of participants’ experiences, perceptions, and attitudes related to the research topic.

All participants were emailed a participant information sheet before attending focus groups and provided informed consent. Focus groups were conducted in person (Phase 1, audio recorded) or by Zoom (Phase 2, audiovisual recording) and were led by staff external to the anatomy team to avoid influencing responses. Recordings were then transcribed and anonymised by a third-party transcriber (Phase 1) or by Descript software(Phase 2) (Descript, Inc., 2017), checked by a staff member who did not know the students.

Focus group questions were decided by the anatomy team to explore issues surrounding the Anatomage tables commonly addressed in class (Supplementary Tables 1, 2 and 3). The protocol was designed to elicit rich and diverse responses, ensuring comprehensive coverage of the research themes.

#### Thematic analysis

Researchers familiarized themselves with the data through multiple readings of the transcripts. This initial immersion allowed for an in-depth understanding of the content and context.

All the researchers are specialised anatomy educators without a clinical background but extensive experience in teaching across multiple clinical programmes. They have been using Anatomage for many years and so have written the research questions and analysed data with that positionality. The questionnaire and focus group questions were decided collaboratively amongst the anatomy team in the phase one and phase two data collection.

Initial codes were generated through a systematic process of line-by-line coding, identifying patterns, concepts, and recurring ideas within the data. Coding was both deductive, guided by predetermined research questions, and inductive, allowing for the emergence of unexpected themes. In Phase 1 three people carried out focus group coding and in Phase 2 two people completed the coding. Multiple coders were involved to minimize bias and collaborate on the development of themes from these data. During Phase 1 Nvivo software (Denver, Colorado) was used to code the transcripts, and Microsoft Word in Phase 2 (Redmond, Washington); this disparity was due to changes in staffing during the process. This change in staffing also meant the people coding the data changed, which may have led to different interpretations of the data.

Codes were grouped into potential themes based on shared characteristics or meanings. Themes were refined through an iterative process of constant comparison, ensuring coherence and relevance to the research objectives. An iterative process of constant comparison between codes and themes contributes to the validity of the analysis.

Themes were reviewed, refined, and adjusted as needed through discussions among the research team. These were conducted to critically examine assumptions and interpretations, contributing to the study’s rigor a collaborative approach enhanced the rigor and validity of the thematic analysis. The researchers critically examined and disclosed their own positionality and perspectives related to the research topic. As anatomy educators without clinical backgrounds but with extensive experience teaching with technology, the researchers were conscious of how their background shaped research questions and interpretations. During data analysis, the researchers engaged in continual self-reflection about their theoretical and personal assumptions, preconceptions and biases.

Thematic analysis served as a robust methodological framework, allowing for a systematic exploration of qualitative data and providing valuable insights into the research objectives. The rigor and ethical considerations embedded within the study design contribute to the trustworthiness of the findings, ensuring that the themes identified authentically represent the perspectives and experiences of the study participants.

#### Quantitative data collection

For the qualitative data collection, first year BMBS students were asked to fill out an online questionnaire using Microsoft Forms. The feedback form was optional and anonymous as students did not need to provide any identifying information. Students were asked to complete the form at the end of an Anatomage session and were also told about the research project and its aims at determining how to improve Anatomage experiences in the future.

Likert scales were used to explore the views of various TAM factors including perceived usefulness, perceived ease of use, time, self-efficacy, self-expectations of using the Anatomage table, perceived peer expectations of using the Anatomage table, enjoyment, working with friends, clear instructions, content support, technical support and time (Supplementary Table 4). 65 students opted to fill out the feedback form. Statistical analyses were conducted using IBM SPSS Statistics 25 (IBM Corp., Armonk, NY).

## Results

### Qualitative data

Reflexive thematic of the first phase of focus groups produced four key themes; the educational value of the table, learning to use the table, groupwork (explored in a separate paper) and prioritisation of time.

#### Educational value of the table

“Cos it is it’s great, like you said it’s an incredible piece of technology, it is human anatomy when we want it…​” Person 1, Phase 1, Year 1

“To use it as a learning resource to facilitate our learning and to apply our learning, the Anatomage table is great​” Person 2, Phase 1, Year 1

Student commented positively on the educational value of the table in the first phase focus groups. There was recognition of the benefits of digital cadavers, such as the ability to undo mistakes, the opportunity to move the cadaver around to identify the route of a vessel from the anterior to posterior compartment of the limb, and the ability to relate to medical imaging on the same screen.

#### Learning to use the table

While students in the first phase of focus groups were enthusiastic about the potential the Anatomage tables had to support their learning, it was clear that the initial training provided did not prepare the students to use the system.

“I think it definitely is amazing, but quite honestly I feel so quite wary of it cos I don’t know how to use it…” Person 4, Phase 1, Year 2

“At the very first week of uni they gave us all sheets and then put us into our groups of five and told us to come in and just learn how to use it on our own, and like press this and press that, we were so lost…” Person 7, Phase 1, Year 1

There was an assumption that students, as ‘digital natives’ would feel more comfortable using this new technology than the staff. This failed to take into consideration the impact of existing anatomical expertise, that allowed staff to focus solely on working the technology. While students had to both understand the anatomy and the technology. This increased cognitive load reduced the efficacy of the learning opportunity.

“…so if we had a session about Anatomage teaching that was compulsory then maybe it could be applied and we would know how to book, how to use it, and then understanding how to use it then we would be more inclined to book and use it as consolidation.” Person 3, Phase 1, Year 2

As such, subsequent inductions built on prior learning of etymology and bony anatomy to help the students assemble the major vessels of the vascular system, by understanding how their names typically relate to their location or region they supply. This scaffolding approach reduced cognitive load, allowing students to focus on using the system.

The lack of facilitator in initial inductions also formed a barrier, particularly if students met technical issues or had questions that they could not resolve. For example, if the previous group had closed the application, or students used a different functionality and didn’t know how to return to the original pre-set to work through the induction material.

“The more often you use it, the more you get used to it, but if it doesn’t work for you the first few times you’re just going to get sick of it and you’ll just go I’d rather not use it.​” Person 4, Phase 1, Year 1

A new compulsory facilitated induction was designed for the students to learn how to use the Anatomage table at the beginning of Year 1. This introduced students to the primary basic table functions used in class, highlighted groups roles to encourage engagement, using an accompanying iPad and the various bodies available to access on the table.

In the second phase focus groups students reported having a better basic understanding of how the Anatomage table worked and expressed a desire to learn more about the Anatomage tables different functions.

“Yeah, it would be nice, maybe, to a bit more detailed on how it works, because we only really click through like the different presets” Person A, Phase 2

As a result, the number of tasks were reduced and time was provided to test the different functions of the table, and compare the different cadavers, to identify healthy and pathological variations.

#### Prioritisation of time

“In first year quite honestly you’re new to uni, you want to give everything a go, learning how to use the Anatomage table in your free time is not top of your priority list…​” Person 3, Phase 1, Year 2

Students have many demands on their time, and so, expectations of students visiting the teaching facilities to use the Anatomage system for self-directed study were, perhaps, idealistic. However, the tables remain available for students and societies to use outside of teaching sessions. Although, if the tables are switched off and no staff are available to help, this would form a barrier to use. As such, Technical Assistants, students employed to provide support for students to use the teaching rooms out of hours, are trained to provide Anatomage support.

“I think if they gave us the homework sessions, we’d be using it more often.​” Person 1, Phase 1, Year 1

“I think instructions on how to actually set up by yourself and maybe like a, uh, a key of which, uh, icon does, what would probably also help out” Person 1.3, Phase 2

Multiple students mentioned that they wanted more structured sessions, some referring to “homework” sessions around the Anatomage tables encouraging them to come and use the tables but also tell them how to use it. As a result, posters welcoming users with a quick-start guide would also make the tables more accessible. Furthermore, Anatomage quiz consolidation sessions were created. These are run by the anatomy team and students don’t have to worry about how to use the Anatomage as they come in and the activity is set up for them. While this does not encourage students taking the lead on the machine it is hoped it will build confidence in the cohort.

Access to Anatomage is particularly difficult for students on placement or those that live a distance away. Remote access to the tables could address this issue, however, with virtual anatomy, it is essential to choose the correct platform for the job. During the COVID-19 pandemic, students were provided free access to the Complete Anatomy app. Students have engaged well with this easily accessible resource.

“I think it’s because it’s [Complete Anatomy] just more easily available…compared to the Anatomage tables.” Person 1.3, Phase 2

The idea of convenience was mentioned multiple times, this likely due to the fact students need to attend university to use the Anatomage tables. As such, the anatomy team has utilised Complete Anatomy for preparatory and consolidation activities for sessions. Although Anatomage is used for preparatory tasks that are beyond the scope of the Complete Anatomy application.

### Quantitative results

#### Subjective norm

Subjective norms were measured using two questions, whether students thought they should be using the Anatomage table in their SDL (M = 7.61, SD = 1.89) and whether they thought their peers expected them to be using the Anatomage table during SDL (M = 4.95, SD = 2.62).

In the multiple linear regression model, enjoyment had the greatest influence on perceived usefulness, followed by self-expectation.

Correlational analyses between ratings suggest that students who thought that they should be using the Anatomage table during their SDL time also thought using the Anatomage was easier than others (*r* = 0.320, *p* < 0.05, Table 1). These students also perceived the Anatomage as more useful (*r* = 0.510, *p* < 0.005, Table 2). Students who thought that their peers expected them to use the Anatomage during SDL also reported finding the Anatomage table easier to use (*r* = 0.408, *p* < 0.005, Table 1) and more useful (*r* = 0.395, *p* < 0.005, Table 2).

**Table 1:** Correlations between external factors and the perceived ease of use are represented by their *r* values and associated *p* values, following Spearman’s rank corelation coefficient analysis. Correlation coefficients between perceived ease of use and external factors are represented by the β value and associated *p* values following linear regression analysis

	Correlation		Regression	
	*r*	*p*	β	*p*
External variables				
Enjoyment	0.550	< 0.001	0.402	< 0.001
Self-efficacy	0.448	0.020	0.255	0.018
Social Norms				
Peer expectations	0.408	0.001	0.228	0.034
Self-expectation	0.320	0.009	NSD	NSD

*NSD* no significant difference

**Table 2:** Correlations between external factors and the perceived usefulness are represented by their *r* values and associated *p* values, following Spearman’s rank corelation coefficient analysis. Correlation coefficients between external factors and the perceived usefulness are represented by the β value and associated *p* values following linear regression analysis

	Correlation		Regression	
	*r*	*p*	β	*p*
External variables				
Enjoyment	0.806	< 0.001	0.702	< 0.001
Self-efficacy	0.287	0.020	NSD	NSD
Social Norms				
Peer expectations	0.395	0.001	NSD	NSD
Self-expectation	0.510	< 0.001	0.324	< 0.001

*NSD* no significant difference

In the multiple linear regression model the order of causation influence on ease of use was as follows from most to least: Enjoyment, Peer expectation, Self-efficacy.

Where ease of use was the outcome variable, peer expectation was the third most influential factor in determining it (β = 0.228, *p* < 0.05, Table 1). Self-expectation of use was the second most influential factor in determining usefulness (β = 0.324, *p* < 0.005, Table 2). Taken together we can determine that the subjective norm is a driver in determining ease of use and perceived usefulness of the Anatomage table.

#### Enjoyment

Students were asked if they enjoyed using the Anatomage table (M = 7.82, SD = 1.53). Enjoyment was positively correlated with both perceived ease of use (*r* = 0.550, *p* < 0.005, Table 1) and perceived usefulness (*r* = 0.806, *p* < 0.005, Table 2). In a multiple linear regression, enjoyment was shown to have the biggest influence over both perceived ease of use (β = 0.402, *p* < 0.005, Table 1) and perceived usefulness (β = 0.702, *p* < 0.005, Table 2).

**Fig. 5:**
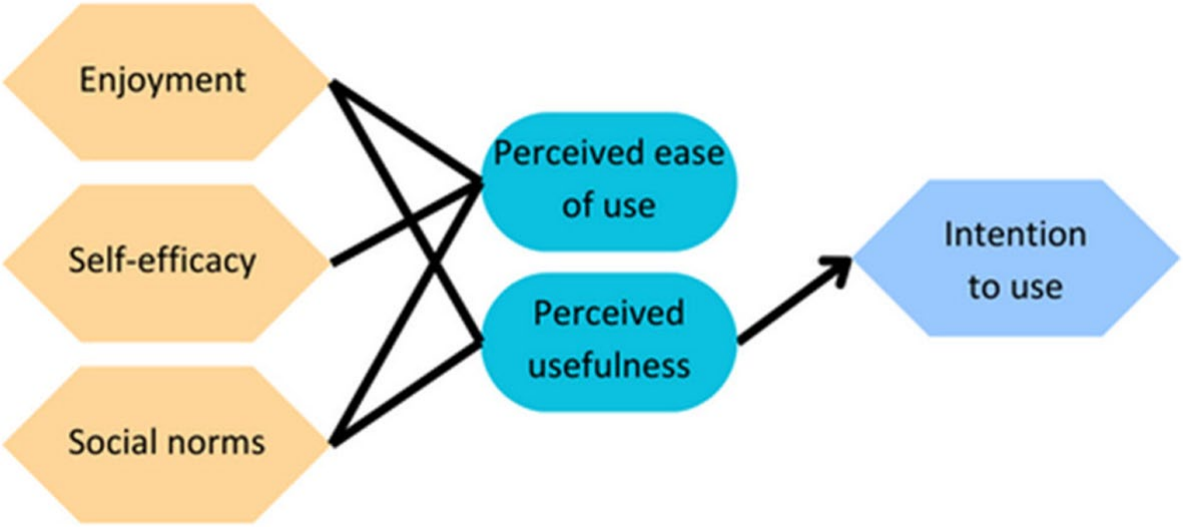
TAM model for Anatomage at Peninsula. These findings resulted in a different TAM model for the Anatomage table than the original, these are summarised in the following figure. It was identified that subjective norm and enjoyment directly influenced both perceived usefulness and perceived ease. Self-efficacy was correlated with perceived ease of use. These results did not find that perceived ease of use had a significant effect of intention to use, however, perceived usefulness did. This study did not extend to look at actual use

It is therefore imperative that students enjoy their experience when using the Anatomage table or any virtual anatomy platform if they are going to adopt it (Fig. 5). However, data on what makes using the Anatomage table an enjoyable experience was not collected in this study.

#### Self-efficacy

Students were asked how good they thought they were at using technology using a Likert scale of 1–10 (M = 7.8, SD = 1.80) Self-efficacy ratings were positively correlated with ease of use (*r* = 0.448, *p* < 0.005, Table 1). In a linear regression model self-efficacy was the second most important factor in determining ease of use (β = 0.255, *p* < 0.05, Table 1). This result indicates that students who think they are good at using technology in general also perceive the Anatomage table as easier to use (Fig. 5). Self-efficacy was also positively correlated with perceived usefulness (*r* = 0.287, *p* < 0.05, Table 2), indicating that students who thought they were better at using technology also perceived the table as more useful.

Furthermore, it was also observed that ease of use was correlated with usefulness (*r* = 0.495, *p* =  < 0.005). Indicating that those who perceived it easier as to use also perceived it to be more useful, a result seen in other publications [29].

#### Intention to use

Students were asked if they intended to use the Anatomage table in the future to capture intended use. Students could respond: Yes, for SDL and extracurricular activities, Yes, for SDL or extracurricular activities, Maybe, or No. These responses were then scored from 1, for ‘no’ through, to 4, for ‘Yes, for SDL and extracurricular activities’.

Spearman rank correlation coefficient scores showed that intention to use was positively correlated with perceived usefulness (*r* = 0.481, *p* < 0.005). However, ease of use did not significantly correlate with intention to use. This result does not fit with previously suggested TAM models.

Multiple linear regression was conducted predicting intention to use from perceived usefulness and ease of use. Whilst perceived usefulness was a significant predictor of intention to use (β = 0.067, *p* < 0.005), ease of use was not. This result could possibly be explained by the fact that medical students are a self-selecting group, of highly motivated ambitious students who do not prioritise ease of use as an important factor in their acquisition of knowledge.

One of the limitations of this study is that it did not extend to look at actual use, future research could explore the link between intention to use and actual use before and after the external factors were manipulated. However, the purpose of this study was to identify factors which will influence ease of use and perceptions of usefulness.

## Discussion

Medical education has witnessed a transformative shift with the integration of advanced technologies, such as the Anatomage table, into anatomy teaching [4, 30, 31]. The adoption of these virtual anatomy platforms holds significant promise for enhancing student learning experiences. In the context of this study, the focus was on addressing student concerns regarding the effective utilization of the Anatomage table, ultimately aiming to optimize its integration into the medical curriculum and produce a framework others could use for integrating technology into their health degrees.

Thematic analysis of the first-phase focus group feedback revealed a common concern among students: a lack of familiarity with the Anatomage table, leading to underutilization and missed learning opportunities, this reflects findings from previous research [32]. Recognising the importance of addressing this issue, a student-focused induction was implemented. This intervention aimed not only to alleviate initial apprehensions but also to foster a more comprehensive understanding of the technology.

Interestingly, the second-phase focus group discussions indicated a shift in student focus from a lack of knowledge about the Anatomage table to a desire for deeper insights into its functionalities. While this shift suggested a positive response to the induction, it also prompted the need to explore factors influencing students’ intention to engage further with the technology. Despite the relatively small sample size in the current work, the findings replicate those of others [32], and as such sample bias or lack of representation are less of a concern, and the findings from phase one regarding concerns about using the Anatomage appear reliable.

To delve deeper into the determinants of student acceptance and use of the Anatomage table, the study employed the TAM. TAM, widely recognised in various technological contexts, provided a theoretical framework to assess the perceived ease of use and perceived usefulness of the Anatomage Table [17, 20, 21]. Previously, a study by Fyfe et al., had determined that students view the Anatomage as the least useful tool for learning in comparison to plastinates, videos and models [32]. However, this runs counter to the evidence that the addition of technology to anatomy teaching increases understanding [4]. As digital anatomy adjuncts are shown to be beneficial this suggests a need to determine further why students do not always agree.

Building upon previous TAM studies that highlighted the role of enjoyment, the current study identified it as a critical factor influencing students’ perceptions [19]. However, the specific aspects contributing to student enjoyment were not investigated. However, game-based learning has the potential to enhance enjoyment, this study suggests the exploration of gamification strategies, leveraging the Anatomage table’s quiz modalities to make learning a more engaging experience [33, 34]. Some recent studies in anatomy and medical education have shown that gamification has the potential to improve medical students’ academic performance and increase engagement and motivation, indicating multiple potential benefits to employing gamification as a strategy to successfully embed technology into the curriculum [35, 36].

Another noteworthy finding was the significant impact of student self-efficacy on perceived ease of use which agrees with other studies. With the increasing reliance on computer software in education, it becomes imperative to identify and support students with lower self-efficacy. Identifying these students could be done through a survey which included questions such as how good do you think you are at using technology? When did you first own a computer? The age at which students first own a computer has been shown to be inversely correlated with higher self-efficacy and technology and therefore an important question to ask students [37]. Another way to improve confidence and therefore self-efficacy would be to provide peer to peer teaching with any technology that was being embedded into the curriculum. A study by Banjoko et al., determined that peer led teaching on the Anatomage increased student confidence suggesting a clear benefit to employing this method [38]. Furthermore, near peer teaching has shown to be a powerful tool in anatomy education and medical education, carving an important role in students’ education [39, 40]. At Peninsula our Year 5 anatomy demonstrators have been running Anatomage sessions to capitalise on this effect, however, as this pilot started this academic year, there is currently no research to establish the effect of this intervention.

**Fig. 6:**
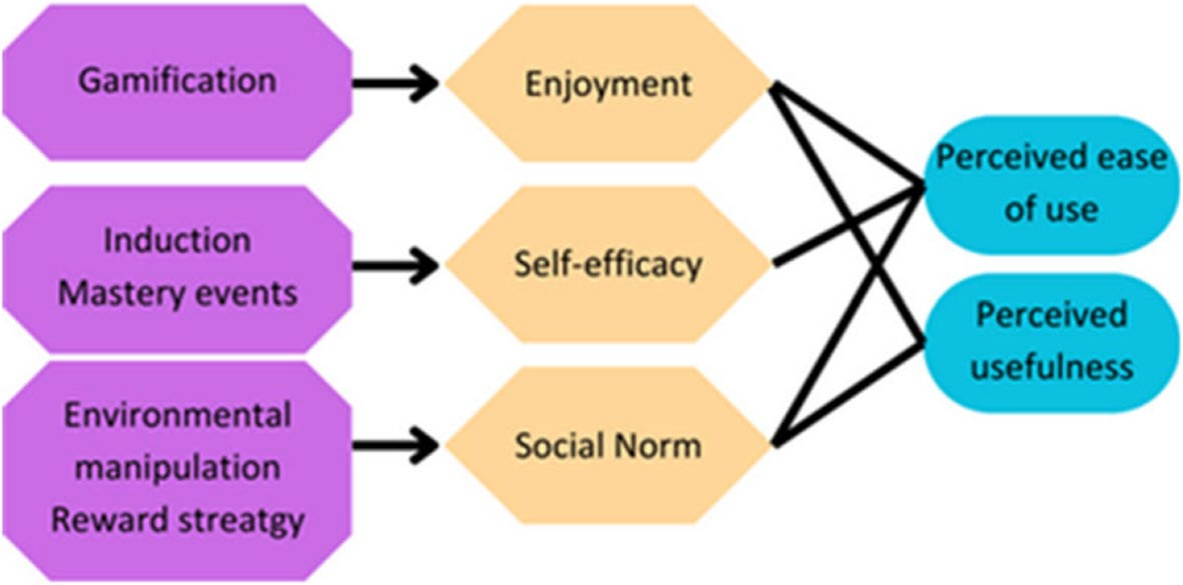
Suggested methods to increase student use of technology. This research demonstrated that enjoyment had the biggest influence over perceived ease of use and usefulness and therefore a method to increase enjoyment would be to gamify the use of any technology being embedded into the curriculum. Self-efficacy was also an important factor, ways to increase self-efficacy are potentially through inductions and through mastery events where they learn how to use the technology and experience high levels of success while doing so. To change the social norm to encourage technology use, environmental manipulation can be used such as withholding information that can only be retrieved by interacting with the technology or using a reward system which encourages people to interact with the technology

Mastery events have also been shown to have a prominent influence in reinforcing or changing self-efficacy perceptions, these mastery events are when students are exposed to the activity and experience success or failure in what they set out to do [41]. In terms of embedding technology into the curriculum this means the first few times a student interacts with a technology need to be successful. The Anatomage induction served as an initial step, but additional easy sessions with high success rates may be necessary to ensure that all students, regardless of their self-efficacy levels, can fully leverage technology for learning (Fig. 6).

The study also highlighted the influential role of social norms in determining actual use. Recognizing that students may be influenced by the expectations of their peers, the study suggests strategies to alter social norms. Environmental manipulation, where information is accessible only through interaction with the Anatomage table, and a reward approach, involving praise for completing tasks using virtual anatomy platforms, are proposed as means to influence both extrinsic and intrinsic motivations [42]. However, one limitation around environmental manipulation remains and that is that students must come into the facility to use the Anatomage table. Potential ways to overcome this barrier are to organise events when students are on campus for them to use the Anatomage tables, another way is that the Anatomage table does offer remote access using a PC. However, this comes with other limitation such as the need for the Anatomage table to be on and limited access in terms of student numbers as only the person with the PC can access the Anatomage table software. When considering technology implementation into any curriculum it is therefore imperative to determine student accessibility to the technology. At Peninsula we have promoted the reward approach by developing the Peninsula Anatomy Award. A certificate students can achieve by attending at least six extracurricular sessions on the Anatomage table. These Anatomage sessions use quiz modalities whereby students play against one another trying to correctly identify different structures. These sessions are located at the end of each two-week case unit and focus on the region or system they have just learned. If they obtain this award, they are then able to compete in the Peninsula Anatomy Championships where they compete against one another to win a prize for first, second or third place. While we have yet to determine the effect of this pilot, others have reported that introducing a reward improves outcomes in exams, suggesting increased engagement in anatomy [43]. It is hoped that this approach will increase intrinsic motivation of students to come in and use the Anatomage tables, while also addressing students’ suggestions of providing more “homework”.

Despite following best practice for this research approach there will be some limitations of these data. As focus groups, by definition, are a smaller group than the population studied, there is the chance that their views were not fully representative of the entire cohort. The student cohort also changed during the duration of the study, as they progressed through the programme, and as such it was not possible to test the interventions on the original cohort.

Qualitative data analysis can be subjective; however, these effects are minimised by multiple coders. This included a change in coders between phase 1 and 2, due to researchers going on maternity leave. Analysis was carried out with NVivo only in phase one, due to the software being unavailable at the time of phase 2 analysis. The Covid-19 pandemic also interrupted this research as students were unable to access to Anatomage table during lockdowns.

This study did not test whether actual use did correlate to perceived ease of use or usefulness, as this had already been identified in many other studies [20, 22, 29, 44]. However, due to the novel technology used in this study this should be further investigated. Another limitation of this study is that it was not analysed whether any of the recommendations altered enjoyment, social norm or self-efficacy. While previous literature informed these recommendations student cohorts are constantly changing and evolving, and this needs to be reflected in research to ensure that current recommendations are still applicable.

In conclusion, the study underscores the need for a comprehensive, multi-aspect approach to successfully integrate virtual anatomy platforms and technology into the medical education curriculum. By addressing practical concerns, enhancing enjoyment, and reshaping social norms, educators and institutions can create an environment conducive to the optimal use of advanced educational technologies. Future research should explore the long-term impact of these interventions and consider expanding the scope to other technological tools in medical education, contributing to the broader discourse on effective technology adoption in the medical field.

### Supplementary Information

**Additional file 1:** Questions asked in student focus groups. The questions listed were those specifically relating to the Anatomage table.

## Data Availability

The datasets used and/or analysed during the current study are available from the corresponding author on reasonable request.
