# Supplementary material for: Fostering engagement in virtual anatomy learning for healthcare students
Source: BMC Med Educ. 2024 Apr 16;24:414. doi: 10.1186/s12909-024-05278-5 (PMC11020670; doi:10.1186/s12909-024-05278-5)
Supplement: Supplementary file 1 — Additional file 1: Questions asked in student focus groups. The questions listed were those specifically relating to the Anatomage table. [file 12909_2024_5278_MOESM1_ESM.pdf]

Questions Phase 1 Year 1
Thinking about all you need to know to be a good clinician in the future, how important is anatomy?
Are there different learning approaches that you use specifically for anatomy?
Any comments on how you use the Anatomage table and how useful it is?
What are other people's experience of using Anatomage?
Have there been instances where the Anatomage table has worked particularly well?
You described that you forget how to use the Anatomage table between sessions. How do you think this could be resolved?
What happened in the first year then when you were first introduced to the Anatomage table?
Do you have any final thoughts about the Anatomage table?

Supplementary Table 1: Questions asked to Phase 1 Year 1 focus groups. Questions are listed which were asked by the facilitator to the Year 1 BMBS students. The questions are listed which specifically applied to the Anatomage table.

Questions Phase 1 Year 2
Thinking about all you need to know to be a good clinician in the future, how important is anatomy?
How have you found learning anatomy compared to your other subjects?
Do you have any final thoughts about the Anatomage table?

Supplementary Table 2: Questions asked to Phase 1 Year 2 focus groups. Questions are listed which were asked by the facilitator to the Year 2 BMBS students. The questions are listed which specifically applied to the Anatomage table.

Questions Phase 2
Can you think of a particular time you used the anatomage table? What about it worked well, or not so well?
Previously students were worried about using the anatomage table and suggested a more thorough induction. Do you think the new induction you had helped you use the table?
When you're using the Anatomage table, do you remember that the cadavers were real people? Does this affect how you interact with the activities?
Do you feel that using the anatomage table in your SDL would be helpful?<br>1. What are the barriers that stop this happening?
What support could be offered to remove any barriers to using the anatomage table?

Supplementary Table 3: Questions asked in Phase 2 focus groups. Questions are listed which were asked by the facilitator to the Year 1 and Year 2 BMBS students along with Year 1 and Year 2 Radiography students. The questions are listed which specifically applied to the Anatomage table.

Questions	Scale
How easy do you think the Anatomage table is to use?	1-Not at all easy, 10-Very easy to use
How useful do you think the Anatomage table is in your education?	1-Not very useful, 10-Very useful
How good/competent do you think you are at using technology? (just general technology i.e. your phone, computers etc.)	1-Not at all good, 10-Very good
Do you enjoy using the Anatomage table?	1-Strongly dislike using it, 10-Really enjoy using it
How much do you agree with this statement? "I should use the Anatomage table during my SDL"	1- Completely disagree, 10-Completely agree
How much do you agree with the following statement "Having clear prescriptive instructions is important when using the Anatomage table"	1-Completely disagree, 10-Completely agree
How much do you agree with this statement? "My peers expect me to use the Anatomage table for my SDL"	1-Completely disagree, 10-Completely agree
How much do you agree with the following statement "On hand technical support is important when using the Anatomage table"	1-Completely disagree, 10-Completely agree
How much do you agree with the following statement "Having plenty of time to use the Anatomage is important when using it"	1-Completely disagree, 10-Completely agree
How much do you agree with the following statement "Working with friends is important when using the Anatomage table"	1-Completely disagree, 10-Completely agree
Do you intend to use the Anatomage table in the future in SDL or by coming into extra curricular staff led dissections?	Yes - SDL and extracurricular activities<br>Yes- extracurricular activities<br>Yes - SDL<br>Maybe<br>No

Supplementary Table 4: Questionnaire for quantitative feedback. This questionnaire was completed by Year 1 BMBS students following an extracurricular session on the Anatomage table. The questionnaire asked the questions detailed in the table and was completed on Microsoft Forms.
